# EVALUATION OF USABILITY OF A NEONATAL HEALTH INFORMATION SYSTEM ACCORDING TO THE USER’S PERCEPTION

**DOI:** 10.1590/1984-0462/;2019;37;1;00019

**Published:** 2018-12-13

**Authors:** Lucio Padrini-Andrade, Rita de Cássia Xavier Balda, Kelsy Catherina Nema Areco, Paulo Bandiera-Paiva, Marynéa do Vale Nunes, Sérgio Tadeu Martins Marba, Werther Brunow de Carvalho, Ligia Maria Suppo de Souza Rugolo, João Henrique Carvalho de Almeida, Renato Soibelmann Procianoy, José Luiz Muniz Bandeira Duarte, Maria Albertina Santiago Rego, Daniela Marques de Lima Mota Ferreira, Navantino Alves, Ruth Guinsburg, Edna Maria de Albuquerque Diniz, Juliana Paula Ferraz dos Santos, Daniela Testoni, Nathalia Moura de Mello e Silva, Maria Rafaela Conde Gonzales, Regina Vieira Cavalcante da Silva, Jucille Meneses, Walusa Assad Gonçalves-Ferri, Ricardo Perussi-e-Silva, Olga Bomfim

**Affiliations:** a Universidade Federal de São Paulo, São Paulo, SP, Brasil.; b Universidade Federal do Maranhão, São Luís, MA, Brasil.; c Universidade Estadual de Campinas, Campinas, SP, Brasil.; d Universidade de São Paulo, São Paulo, SP, Brasil.; e Universidade Estadual Paulista “Júlio de Mesquita Filho”, Botucatu, SP, Brasil.; f Instituto Fernandes Figueira/Fundação Oswaldo Cruz, Rio de Janeiro, RJ, Brasil.; g Universidade Federal do Rio Grande do Sul, Porto Alegre, RS, Brasil.; h Universidade do Estado do Rio de Janeiro, Rio de Janeiro, RJ, Brasil; i Universidade Federal de Minas Gerais, Belo Horizonte, MG, Brasil.; j Universidade Federal de Uberlândia, Uberlândia, MG, Brasil.; k Faculdade de Ciências Médicas de Minas Gerais, Belo Horizonte, MG, Brasil.; l Hospital Estadual de Sumaré, Sumaré, SP, Brasil.; m Hospital Geral de Pirajussara, Taboão da Serra, SP, Brasil.; n Hospital Estadual de Diadema, Diadema, SP, Brasil.; o Universidade Estadual de Londrina, Londrina, PR, Brasil.; p Universidade Federal do Paraná, Curitiba, PR, Brasil.; q Instituto de Medicina Integral Professor Fernando Figueira, Recife, PE, Brasil.; r Universidade de São Paulo, Ribeirão Preto, SP, Brasil.; s Rede Brasileira de Pesquisas Neonatais, Rio de Janeiro, RJ, Brasil.

**Keywords:** Medical informatics, Information systems, Health information systems, Systems analysis, Information science, Health personnel, Informática médica, Sistemas de informação, Sistemas de informação em saúde, Análise de sistemas, Ciência da informação, Profissional de saúde

## Abstract

**Objective:**

To measure the level of satisfaction regarding the usability of a neonatal health information system and identify if demographic factors can influence the usability of a health information system.

**Methods:**

A cross-sectional, exploratory study was carried out with a convenience sample of 50 users of the Brazilian Neonatal Research Network. The instrument chosen for the usability evaluation was the System Usability Scale between February and March 2017. The statistical analysis of the collected variables was carried out in order to describe the sample, to quantify the level of satisfaction of the users and to identify the variables associated with the level of satisfaction.

**Results:**

The female gender represented 75% of the sample. The mean age was 52.8 years; 58% had a doctoral degree, average time of graduation was 17 years, with area of practice in medicine (neonatology), with intermediate knowledge in computer science (74%) and mean system use time of 52 months. Regarding usability, 94% rated the system as “good”, “excellent” or “better than imaginable”. The usability of the system was not associated with age, gender, education, profession, area of practice, knowledge in computer science and time of system use.

**Conclusion:**

The level of satisfaction of the computerized health system user was considered good. No demographic factors were associated with the satisfaction of the users.

## RESUMO

**Objetivo:**

Mensurar o grau de satisfação de profissionais de saúde quanto à usabilidade de um sistema de informação em saúde neonatal e identificar os fatores que podem influenciar na satisfação do usuário frente à usabilidade.

**Métodos:**

Estudo transversal e exploratório realizado com 50 profissionais de saúde integrantes dos centros da Rede Brasileira de Pesquisas Neonatais. Para avaliação da usabilidade foi utilizado o instrumento System Usability Scale entre fevereiro e março de 2017. Realizou-se a análise estatística descritiva e inferencial das variáveis coletadas, com a finalidade de descrever a amostra, quantificar o grau de satisfação dos usuários e identificar as variáveis associadas ao grau de satisfação do usuário em relação à usabilidade.

**Resultados:**

Da população avaliada, 75% era do sexo feminino, com idade média 52,8 anos, 58% com pós-graduação (doutorado); tempo médio da última formação de 17 anos; área de atuação em medicina (neonatologia), grau intermediário de conhecimento em informática e tempo de utilização média do sistema de 52 meses. Quanto à usabilidade, 94% avaliaram o sistema como “bom”, “excelente” ou “melhor impossível”. A usabilidade do sistema não foi associada a idade, sexo, escolaridade, profissão, área de atuação, nível de conhecimento em informática e tempo de uso do sistema.

**Conclusões:**

O grau de satisfação do usuário do sistema informatizado de saúde foi considerado bom. Não foram identificados fatores demográficos que influenciassem sua avaliação.

## INTRODUCTION

In a health care environment, decisions are based on factors and indicators obtained through information coming from records of patients assisted at the unit. The storage of clinical, demographic and financial data of the patients is more feasible and accessible to several Brazilian institutions, both public and private.^1^ ^,^ ^2^ ^,^ ^3^

An information system is not only about information technology; but also about the context in which it is inserted, and the profiles of its users, both for the collection and the analysis of the data.^2^ The evaluation of information systems is a necessity for the administrator, both to improve and to justify the high investments in informatics. Nowadays, there is no global measure that clearly shows its use. Therefore, the matter to be studied has to be predefined according to the research object.^4^ ^,^ ^5^

Researchers from the information system field emphasize the need for more dynamic and consistent measurements for the evaluation.^6^ ^,^ ^7^ ^,^ ^8^ To design systems with good usability, the experts need:

- To understand the factors (psychological, ergonomic, organizational and social factors) that determine how people operate and use the computers effectively.
- To translate this understanding into the development of tools and techniques that can help the project.
- To use tools to reach efficiency, effectiveness and safety in the interaction.

The instrument System Usability Scale (SUS) was developed by Brooke, in 1986, and contains ten questions that aim at measuring the usability of several products and services. Compared to other assessment instruments, SUS is technologically agnostic, so it can be used to assess several products and services, such as websites, hardware, multimodal systems, voice command systems, mobile applications and clinical systems.^9^ ^,^ ^10^ It is a robust and versatile instrument, making the research fast and easy. The instrument generates a single score in a scale that is easy to understand. It is also easy to manage, since it shows good reliability and references that support the interpretation of the score.^10^ Another advantage of SUS is that there are no copyrights, so the cost is accessible.^11^

Considering this scenario, by using uncomplicated tools, which consider the opinion of their users, it is essential to evaluate health information systems; once these are important for the decision-making of administrators and health professionals. In this context, there was a study about the evaluation of a health information managing system maintained in a national research network, with the objective of measuring the level of satisfaction of health professionals regarding the usability of a neonatal multicenter health information system, using the SUS, and of identifying the factors that can influence the evaluation of user satisfaction in relation to usability.

## METHOD

Exploratory, observational, descriptive study, with both quantitative and qualitative approach, and cross-sectional design. The project was submitted to and approved by the Research Ethics Committee (CEP) of Universidade Federal de São Paulo, according to Report n. 940,160, and by the Research Commission of the Brazilian Network of Neonatal Research (CP-RBPN).

The research universe was the database of RBPN. The insertion of patients’ data is performed in the 20 associated centers, located in seven Brazilian states, by the coordinators of each unit. The database was developed in REDCap, hosted in a cloud computing system called InterNuvem, maintained by Universidade de São Paulo.

After an invitation to fill out the electronic questionnaire (SUS), the study included all health professionals, representative of the 20 centers and the coordination of RBPN, with access to the data base of RBPN for insertion, verification and/or correction of data. The study excluded professionals from the information technology field and users who, in spite of being registered, did not access the RBPN system. The data collection was carried out between February and March, 2017, in the website http://redcap.epm.br.

The electronic questionnaire was composed of a single screen, divided in two parts. In the first part, the interviewees filled out an identification form with the following variables: sex, age, maximum schooling, year of conclusion of the maximum school year, profession, field of professional work, level of knowledge in informatics, and approximate time of system use. Based on the questions of the first part of the questionnaire, it was possible to characterize the studied population. The variables “age”, “year of conclusion (schooling)”, and “time of use of the data base)” were numerical, and “sex”, “schooling (maximum level)”, and “level of knowledge in informatics” were categorical. The variables “profession” and “field of professional work” were dissertation.

In the second screen, the users answered the SUS, according to Chart 1.^12^ The ten questions of SUS were graded in a Likert-type scale, from one to five, classified, respectively, as: “strongly disagree”, “disagree”, “do not agree nor disagree”, “agree”, and “strongly agree”. Only the last question, included by the researcher, was a dissertation and not mandatory. The total estimated time to answer the questionnaire was from five to ten minutes. After SUS was filled out, the total score was calculated, and generated a single number. To calculate the score, we first add the score of each item, which contributes to a 1 to 5 scale. For items 1, 3, 5, 7 and 9, the individual score is the grade received minus 1. For items 2, 4, 6, 8 and 10, the contribution is 5 minus the grade received.

The sum of all scores was multiplied by 2.5, and this is how the total value of SUS is obtained.^9^ After the scoring and the calculation of the score, it is possible to classify the evaluated system:^13^ £20.5 (worst case); 21 to 38.5 (poor); 39 to 52,5 (median); 53 to 73,5 (good); 74 to 85,5 (excellent); and 86 to 100 (better imaginable). For example: an evaluation of a specific system received the following scores, beint q1 equivalent to the first question of SUS, and so on and so forth:

**Table:** 

q1	q2	q3	q4	q5	q6	q7	q8	q9	q10
5	2	5	2	5	1	4	1	5	1

Odd questions: (5-1)+(5-1)+(5-1)+(4-1)+(5-1)=4+4+4+3+4=19

Even questions: (5-2)+(5-2)+(5-1)+(5-1)+(5-1)=3+3+4+4+4=18

Expression: sum of the questions x2.5=37x2.5=92,5

Classification: better imaginable

**Chart 1:** System Usability Scale questionnaire translated into Portuguese.

Item	Corresponding item in Portuguese
1	I think that would like so use this product frequently
2	I found the product unnecessarily complex
3	I thought the product was easy to use
4	I think that I would need the support of a technical person to be able to use this product
5	I found the various functions in this system were well integrated
6	I thought there was too much inconsistency in this system
7	I would imagine that most people would learn to use this system very quickly
8	I found the system very cumbersome to use
9	I felt very confident using the product
10	I needed to learn a lot of things before I could get going with this product
Not mandatory	Do you have any complaints and/or suggestions referring to the product?

In this study, the data obtained through the SUS questionnaire were analyzed regarding the descriptive analysis of the variables of the first (demographic) and second parts of the SUS questionnaire; the SUS score, by assessing each one of the ten questions; the value obtained by each respondent; and the acquisition of the global mean of the questionnaires answered; and the factors associated with the characteristics of the participants and the obtained scores.

After data collection, the researcher assessed items of the system referring to the objectives and needs of the users, through a checklist developed by Padovani, in order to highlight the positive and negative aspects of the system.^14^

The categorical variables were described by the absolute (n) and relative (%) frequencies. The numerical variables were tested regarding adherence to normal distribution using the Kolmogorov-Smirnov test, and described by mean, standard-deviation, minimum, maximum, median and interquartile interval. The central tendency and variability measures indicated were, respectively, the mean and the standard deviation for the normally distributed variables, and the median and interquartile interval for those that are not normally distributed.^15^ The associations with one or more numerical variables were tested as to the normal distribution using the Kolmogorov-Smirnov test. The correlation between the SUS score and the numerical variables used the Pearson’s correlation coefficient (when both variables presented with normal distribution) or the nonparametric equivalence testing, Spearman correlation, and analysis of variance, or Kruskal-Wallis for more than two categories, according to the adherence or not to the normal distribution. For the conduction of more analyses, the software MS Excel 2010^®^ and IBM SPSS Statistics 20^®^ were used.

## RESULTS

According to the methodology used, 67 participants were identified. After considering the exclusion criteria, 57 users were selected. Among the eligible ones, seven did not answer the questionnaire. Therefore, at the end of the collection period, we obtained 50 participations (87.7%). The characteristics of the participants are available in Table 1.

**Table 1:** Characteristics of the users of the Brazilian Neonatal Research Network system (n=50).

Age (in years)	
Mean (standard deviation)	52.8 (9.7)
Median (minimum-maximum)	53.5 (35-75)
Sex	n (%)
Female	36 (72.0)
Male	14 (28.0)
Schooling (maximum level)	n (%)
College Education	1 (2.0)
Specialization	10 (20.0)
Master’s degree	10 (20.0)
PhD	29 (58.0)
Last maximum title (years)	
Mean (standard deviation)	17.0 (11.6)
Median (minimum-maximum)	14.5 (1-43)
Profession	n (%)
Nursing	1 (2.0)
Medicine	49 (98.0)
Work	n (%)
Intensive pediatric medicine	1 (2.0)
Neonatology	48 (96.0)
Neonatology and patient safety	1 (2.0)
Level of knowledge in informatics	n (%)
Basic	7 (14.0)
Intermediate	37 (74.0)
Advanced	5 (10.0)
Ignored	1 (2.0)
Time of system use (months)	
Mean (standard deviation)	51.6 (38.9)
Median (minimum-maximum)	42 (3-130)

As to the knowledge in informatics, it was observed that three out of four participants declared being at an intermediate level; and one participant, who did not respond, was classified as ignored (absent information).

Regarding the usability questionnaire, SUS, all participants filled out the questions. The mean of the total score was 73.3; with standard deviation of 13.6; minimum value of 37.5, and maximum of 100.

When analyzed by the Spearman coefficient and the Kruskal-Wallis test, the association between the SUS score and the variables age, schooling, level of knowledge in informatics and time of use showed low correlation, not statistically significant, with p-value=0.220; p-value=0.183; p-value=0.512, and p-value:0.155, respectively.

Table 2 shows that 76% of the participants classified the database as “good” or “excellent”, and 18% as “better imaginable”.

**Table 2:** Classification of the usability by users of the computarized system, according to the System Usability Scale (n=50).

Classification	n (%)	Accumulated %
Poor	1 (2.0)	2.0
Median	2 (4.0)	6.0
Good	26 (52.0)	58.0
Excellent	12 (24.0)	82.0
Better imaginable	9 (18.0)	100.0
Total	50 (100.0)	

## DISCUSSION

The SUS instrument applied to the RBPN information system was efficient to assess the usability through the user’ perception, being classified as good (score 73.3) by 52% of the assessed users. The demographic information of the participants did not influence the evaluation of the system, suggesting that the questionnaire can be applied in a multicenter health information system.

There are several free questionnaires to assess the usability of the systems. Of these, the analyst should know which interface will be measured. Among those that can be applied to any interface, the After Scenario Questionnaire (ASQ), of IBM^®^, presents three questions: the Usefulness, Satisfaction and Ease of Use (USE) has 30 questions; and SUS, ten questions.^11^ To apply any evaluation instrument, it is important to compare its advantages in relation to its disadvantages, besides defining the matter to be assessed according to the analyzed system.

Regarding the advantages, it is possible to mention that SUS is capable of providing the view of the user about the studied object, presenting reliable results, regardless of the system or its tasks.^11^ In this study, the demographic factors (age, sex, schooling, profession, field of work, level of knowledge in informatics, and time of system use) were not associated with the result obtained in the SUS score.

Since its conception, SUS is considered to be unidimensional. However, the factorial analysis showed that SUS has two factors: usability (questions 1, 2, 3, 5, 6, 7, 8 and 9) and learning (questions 4 and 10). Despite the good correlation between the whole questionnaire and the separate items, it presents a low level of correlation if used separately. Therefore, we recommend its traditional use.^16^

In SUS, the structure of the questions presents alternation between the positive and negative items due to the short titles, in order to prevent bias in the responses. The purpose is that the participants really agree or disagree with the questions after reading, and not just impulsively.^17^

According to the relative values obtained in the questions of the instrument (SUS), the structure of the even questions highlights the positive aspects of the system, presents more answers “agree” and “strongly agree”, whereas the odd questions present more relative records in “disagree” and “strongly disagree”. This suggests that participants can, with the questions, assess the characteristics of the system.

In this study, a non-mandatory question was added in the end of the instrument, in which the participant could include comments about the system. Despite not belonging to the original structure, the choice was to use this item, because free final questions are suggested as a possible supplement, once the users like to describe adjectives in an evaluation.^18^

By considering the relative values obtained in the questions, the number of comments about the system and its content and the observations made by the researcher with a check-list, it was possible to observe that the RBPN system presents the five attributes of Nielsen’s attributes of usability: learnability, efficiency, memorability, errors and satisfaction.^19^ ^,^ ^20^

In this sense, by applying the SUS instrument, the usability was assessed to understand the subjective factors that determine the effective use of the system, which may be translated into actions that can improve the experience of human-computer interaction.

The SUS Score, despite showing variation between zero and one hundred, is not a percentage. One study compared the SUS Score to relative values. The 50 percentile, or the median, was determined in 68. Therefore, the RBPN system presented good evaluation from the users, with mean of 73.3 and median of 72.5 points.^13^

The dissertation obtained 12 comments from users, among the 50 participants, referring to the system. Most answers were observations regarding the results produced by the system. Aspects such as the comparison between the participating centers, the construction of graphs and tables, besides some adjustments in the filling out of the forms were mentioned.

Besides the application of the SUS questionnaire, the researcher verified items regarding the objectives and needs of the users through a check-list developed and used by Padovani to assess websites. This instrument can be used, because the insertion of data is carried out through REDCap, whose platform is accessed through a web environment.^14^ The weak points observed regarded the administration of errors, because the system verifies the consistency of the data after the form is fully filled out, and presents several fields; and the impossibility to customize or adapt the pages. The positive highlight is the user support. First of all, the users who are registered receive instructions referring to the definitions used in the system and the filling out of the forms. There is also a link with an explanation in each field regarding the fulfillment of the forms, besides the name and the e-mail of the administrator in the pages of access to the system and the forms.

The results obtained from the application of the SUS questionnaire and the check-list used by the research will help the administrators of the RBPN system to understand the behavior, the opinion, the earnings and the needs of the users, so it can be used as a support material for the decision regarding possible changes and/or updates of technological resources.

There are studies which present other initiatives regarding the evaluation of the health information systems. The evaluation of the impact of an information system requires not only the understanding of the computer resources, but also of the behavioral processes.^21^

The users presented more than 40 months of active use of the studied system, did not report flaws in the access, presented intermediate knowledge of informatics and high level of education. Therefore, the evaluation of the demographic data allowed to get to know the characteristics of the users, and analyze if there were factors that could influence the evaluation of the health information system.

By analyzing the demographic data obtained and the characteristics of the RBPN, it is possible to say that the system used is in accordance with one of the concepts of health information, such as the representation of a situation based on the trinomial health-disease-care, which was selected, summarized and organized based on specific interests of a professional or by an institution, according to the objectives in the production of knowledge.^22^ ^,^ ^23^ Besides, it was possible to assess if the subjective matters could influence on the results of the evaluation. There were no interferences in the vision of the user in the system, but it is not possible to state that there is no such influence in the performance of these tasks.

Actually, the SUS instrument was idealized as quick and dirty, that is, an instrument created for a superficial evaluation, which aims at identifying possible inconsistencies in the system quickly. In case it is necessary to perform a more detailed identification, other instruments and methods should be used.

The health information is one of the fields of knowledge in collective health that considers the social determinations of the health-disease process as, among other functions, the structure of care models that enable to reach autonomy in the development of health actions.^17^ Therefore, for the health professional who defines, manages, and practices care-related interventions, research and/or teaching, the knowledge and application of an instrument to measure the usability of a health information system is important, adding value and use to the information that is collected, and analyzed. The evaluation of usability of the RBPN system showed that professionals who use the system consider it to be adequate, which contributes to improve the reliability in the quality of the inserted data. This is important because this database is the starting point for the development of research in neonatology.
